# Is a View of Green Spaces from Home Associated with a Lower Risk of Anxiety and Depression?

**DOI:** 10.3390/ijerph17197014

**Published:** 2020-09-25

**Authors:** Olta Braçe, Marco Garrido-Cumbrera, Ronan Foley, José Correa-Fernández, Gina Suárez-Cáceres, Raffaele Lafortezza

**Affiliations:** 1 Health and Territory Research, Department of Physical Geography and Regional Geographic Analysis, Universidad de Sevilla, Doña María de Padilla s/n, 41004 Seville, Spain; htr@us.es (O.B.); htr3@us.es (J.C.-F.); gina.suarez.ca@gmail.com (G.S.-C.); 2 Department of Geography, Maynooth University, W23 F2H6 Maynooth, Ireland; ronan.foley@mu.ie; 3 Department of Agricultural and Environmental Sciences, University of Bari Aldo Moro, 70126 Bari, Italy; raffaele.lafortezza@uniba.it; 4 Department of Geography, The University of Hong Kong, Hong Kong, China

**Keywords:** anxiety, depression, urban green spaces, view from home

## Abstract

Although a large body of research supports the theory that exposure to nature results in mental health benefits, research evidence on the effects of having a view of green space from home is still scarce. The aim of the present study is to assess the impact that access to a green space view from home has on anxiety and depression. This is a cross-sectional study extracting data from the “2018 Green Spaces, Daily Habits and Urban Health Survey” conducted in Carmona (Spain). The study included variables on sociodemographic and lifestyle, view of green spaces from home, self-perceived health status, and risk of anxiety and depression measured using the Hospital Anxiety and Depression Scale (HADS). Chi-square tests were used to assess variable’s associations and a multiple linear regression models used to identify the variables explaining the risk of anxiety and depression, taking into account sociodemographic characteristics, frequency of visits and view of green spaces from home. According to our results, adults who enjoy a view of green spaces from home have a lower risk of anxiety and depression.

## 1. Introduction

Exposure to urban green spaces has been associated with enhanced self-perceived mental health [1,2,3,4,5], beneficial effects on psychological restoration [6,7], improved mood [7], stress reduction [8], and a protective role against anxiety and depression in adults [9]. Direct exposure to natural environments can be determined by visits to green spaces [10], residential proximity [11], or greener neighborhoods. However, indirect exposure to nature does not require physical presence and includes the view of nature through a window [12]. The simple act of viewing trees or plants can provide restorative effects [8,13,14,15,16], relieve stress and restore attention levels [17], promote relaxation [18], and improve health over time [19,20].

Research on the beneficial and restorative effects of contact with nature has found that viewing natural settings contributes to reducing emotional stress and mental fatigue [6,8]. Ulrich (1984) demonstrates that patients in hospitals with a view through a window recover faster than those exposed to a view of a brick wall [21]. Gilchrist et al. (2015), in a study of central Scotland (UK), found that workers’ satisfaction with their office view (particularly of trees, lawns and flowering plants) was associated with improved wellbeing [22]. In a similar vein, Tennessen and Cimprich (1995) categorized the view from university dormitory windows into natural scenes (trees, grass and/or bushes) and built views (paths, roads and/or parking lots), finding positive effects from natural views on students’ ability to direct attention, compared to those with less natural or built views [23]. However, another study found that university students with windowed rooms did not perform better, for different tasks, than those living in a windowless room [24].

The most important views in people’s lives are those from home, allowing long-term visual exposure to nature [20]. Kaplan (2001) found how residential views of nature contributed positively to neighborhood satisfaction and well-being in apartment communities in Michigan (USA) [25]. A study by Elsadek et al. (2020) found how, in high-rise buildings in Shanghai (China), windows with green space views contributed to psychological well-being [26]. In Bulgaria, Dzhambov et al. (2018) found beneficial effects from views of green and blue spaces relating to mental health in university students [27]. A study by Vemuri et al. (2011) noted that residents of the Baltimore metropolitan region (USA) with an increased visibility of trees from their home, reported better life satisfaction [28]. Views of nature from home, concluded Kearney (2006), decreased negative perceptions related to residential density and increased neighborhood satisfaction [29]. Honold et al. (2016) reported that residents with views of vegetation from their home had significantly lower hair cortisol levels as a biomarker of chronic stress reduction [20].

Although these studies support the notion that green views result in mental health benefits, research evidence in this area is still scarce and sometimes contested, particularly in relation to specific mental health measures such as anxiety and depression. Our hypothesis is that individuals who enjoy a view of green spaces from their home benefit from a lower risk of anxiety and/or depression. These findings may support urban planners, public health professionals and local authorities to provide citizens with greater opportunities of enjoying views of green spaces allowing fuller contact with nature as a health-enabling resource [30].

## 2. Materials and Methods

### 2.1. Study Area

Carmona is a historical Roman town located 28 km from the city of Seville (Carmona City Council, 2017) in Andalusia, Spain. It is situated at an altitude of 233 m above sea level and has a surface area of 924.6 km2 [31]. It has a population of 28,620 inhabitants comprised of 14,379 men and 14,241 women [32] and is considered a medium-sized town [33]. Located in the county of Los Alcores [34], Carmona is listed in the Register of Landscapes of Cultural Interest of Andalusia. The town is characterized by hills and escarpments and is surrounded by rural landscapes with irrigated crops such as wheat and sunflowers, along with Mediterranean and riverside forests [35]. In our study, green views are considered to be those areas covered mainly by vegetation within the town of Carmona [36], which include publicly available urban parks and gardens specifically characterized by the presence of trees, bushes, lawns, and flowering plants.

### 2.2. Survey

**Figure 1:**
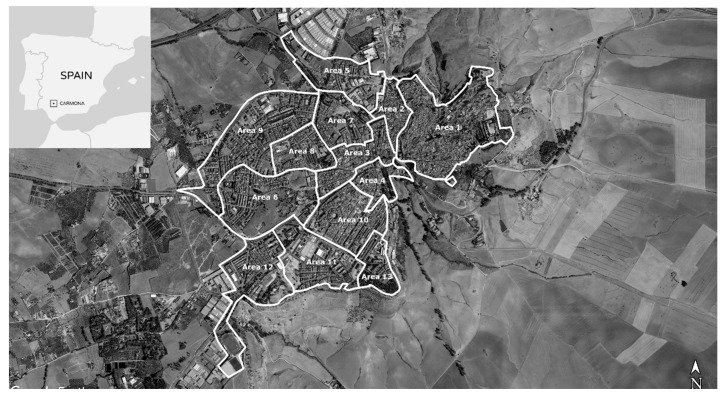
Map of the town of Carmona (Andalusia, Spain) divided into 13 homogeneous areas. Source: Prepared by authors.

Population data were extracted from the “2018 Green Spaces, Daily Habits and Urban Health Survey” that was conducted specifically for this study, led by Health & Territory Research (HTR) of the University of Seville and with the support of Carmona’s city council. The Municipality contributed to the design of the survey and provided four interviewers who were trained by HTR staff [37]. The Municipality has also supported the survey to inform local plans and gain insight into citizens’ preferences on the management and development of green spaces and mobility as public assets within the town. For study purposes, the municipality of Carmona was divided into 13 homogeneous areas through a zoning process taking into account urban and spatial factors and highlighting the predominant urban landscape, household typology (single house, attached, semi-detached, or apartment building), construction date, and presence of infrastructure and services (Figure 1).

**Figure 2:**
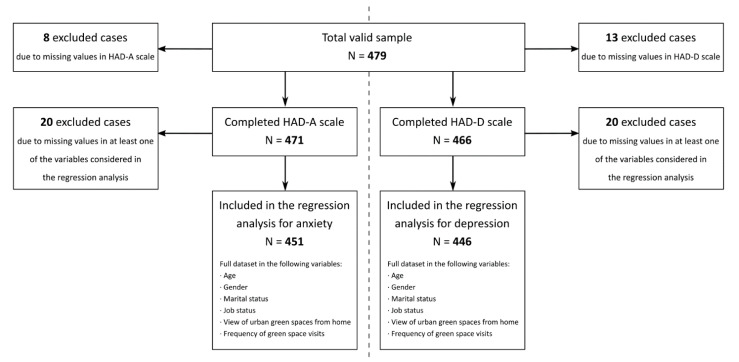
Study sample selection flow chart.

For optimal data collection, door-to-door household surveys were completed during two periods: from February to May and September to November 2018. A representative sample of respondents was obtained by age, gender, and geographic reference data from the Carmona population census updated for 2017 with a 95% confidence level. The inclusion criteria taken into account to evaluate anxiety and depression were respondents older than 18 years of age with at least one year of residence in their current home, established in previous studies [1]; identified as the minimum time required to achieve mental health benefits from a green view. We obtained 479 respondents between 18 and 64 years of age residing in the main family dwelling at the time of the survey (Figure 2).

For this study, the following five sections were considered:

(1) **Sociodemographic variables**: age group (18–31, 32–48, and 49–64 years old); gender (male and female); marital status (married, single, divorced, living as a couple, and widowed); educational level (uneducated, primary school, high school, and university); and job status (employed, unemployed, temporary sick leave, retired, student, and homemaker).
(2) **Lifestyle variables**: smoker (yes/no); alcohol intake (yes/no); physical activity (yes/no); and number of hours of sleep per day (<6 h, 6–7 h, 7–8 h and >8 h).
(3) **Self-perceived health status**: motor impairment (yes/no); and perceived health status over the past year (very good, good, fair, poor, and very poor).
(4) **Anxiety and depression variables**: measured through the Hospital Anxiety and Depression Scale (HADS). HADS is a reliable self-assessment screening instrument for detecting possible cases of anxiety and depression; it consists of seven questions related to anxiety and seven questions related to depression based on the following scoring system: 0–7 = no case, 8–10 = borderline case, and 11–21 = case of anxiety or depression [38,39].
(5) **Green space variables**: view of urban green spaces from home referred to the possibility of viewing green spaces from any of the home windows and was assessed with the following question: “From your home, are you able to see any green space (parks or gardens)?” (yes/no). A second variable recorded frequency of green spaces visits (daily/not daily) with the category not daily composed of “never”, “rarely”, “1–2 days a week”, and “3–4 days a week” options.

The dependent variables used in our study were anxiety and depression measured using HADS, while the independent variable was view of green spaces from home. Other variables were introduced to analyze possible associations with or modifications to the effect of green space views from home on the risk of anxiety and depression.

### 2.3. Statistical Analyses

The frequency, percentage, mean and standard deviation calculations were used to describe the sample. The Chi-square test was used to analyze categorical variables and compare the distributions of anxiety and depression measured by HADS (“No case”, “Borderline case”, and “Case”) with view of green spaces from home (yes/no). The variables: age, gender, educational level, marital status and employment status were introduced into the regression models to predict possible associations with mental health, while the remaining socio-demographic and lifestyle variables were only used to describe the population. In addition, a multiple linear regression model was used to explain the possible relationship between anxiety (numerical scale from 0 to 21) and depression (numerical scale from 0 to 21) based on age (numerical variable), gender (categorical variable taking female as the reference category), marital status (categorical variable taking single as the reference category), educational level (ordinal variable), job status (categorical variable taking unemployed as the reference category), frequency of green space visits (categorical reference taking daily visits as the reference category) and residential view of green spaces (categorical variable taking no views as the reference category).

## 3. Results

Table 1 shows that on average the respondents were 43 years old, with more women than men by a marginal difference. The majority were married, had primary education, were employed, non-smokers, drank alcohol, performed some physical activity, did not frequent green spaces daily, slept for 6–7 h per day, had a good self-perceived health status, and no motor impairment. Across the general population, a significant risk of suffering from anxiety (borderline case 26.5% and case 13.8%) and depression (borderline case 5.8% and case 3.4%) was observed, based on the HADS (Table 1).

The majority of respondents lived in homes without green space views (75.7%). According to the bivariate analysis, there were no significant differences between view of green spaces and anxiety and depression. Even so, the cases of anxiety were more than four times greater than the cases of depression for respondents with no green space view from their home (Table 2).

The variables that explain the risk of anxiety were age (*p* < 0.001), gender (*p* = 0.001), educational level (*p* = 0.035), and view of green spaces from home (*p* < 0.015). Our study finds that older adults, women, individuals with a high educational level, and those who do not enjoy a view of green spaces from home are more likely to be at risk of anxiety (Table 3).

The variables explained by the model were job status (*p* = 0.001) and view of green spaces from home (*p* = 0.013). In our study, unemployed individuals and those who did not enjoy a view of nature from home were more likely to suffer from depression (Table 4).

## 4. Discussion

Our study shows that people who enjoy a view of green spaces from home present a lower risk of anxiety and depression. Although the Chi-square test showed independence between anxiety and depression and views of green spaces, using multiple linear regression identified that higher anxiety and depression levels were associated with the lack of green space views from home. Therefore, our results corroborates previous findings about the mental health benefits of having access to a green view from home [25,26,29].

While some studies have found favorable relationships between generalized exposure to green spaces and anxiety and depression [9,40,41], a specific view from home was not previously considered. Therefore, our research addresses gaps in the literature by exploring the effects of green spaces views on anxiety and depression. Some studies have identified the beneficial effects of green views on mental health [9], and as a potential for restorative care [13,17,42,43]. Wang et al. (2016) found no significant differences for anxiety relief levels in relation to access to a green space view from home, but did so in relation to video scenes [17]. However, the limited number of studies assessing the potential effects of green views from home on specific mental disorders (anxiety and depression) makes further research in this field necessary.

We find that women, older people, and those with a higher level of education are at a greater risk of anxiety; while unemployed people have a higher risk of depression. Considering that anxiety and depression are the most prevalent mental disorders [44], increasing urban greenery by adding plant species (trees, bushes, lawns, and flowering plants) to residential streets should be considered a key intervention aimed at reducing this prevalence. In cities with limited space, green walls and rooftop gardens should be considered as an alternative in the design of more livable urban environments. Recognizing the public health benefits of a green view from home, urban planners should focus not only on increasing the amount of urban greenery, but also on designing greener cities in which more citizens enjoy views of nature from their homes. From a methodological point of view, one of the strengths of this study is the selection of a representative sample of survey respondents by gender, age-group and geographic area. Face-to-face interviews were conducted as this survey method increased the accuracy of the data collected. In addition, the use of the validated HADS scale provided standard and comparable data on anxiety and depression in relation to views of green spaces from home. However, as this is a cross-sectional study the analysis of the results should be interpreted with caution given that exposure and outcome were simultaneously assessed. Finally, the initial hypothesis raised has been corroborated from the proposed analyses and a higher relationship of anxiety and depression has been observed in people who do not have a view of nature. In addition, other factors associated with anxiety and depression such as age, gender, educational level, marital status and employment status have been identified.

### Relevance of the Findings to Policy-makers and Practitioners

These findings should serve as evidence for urban planners, public health professionals and local authorities to provide citizens with the opportunity of enjoying a view of green spaces allowing contact with nature.

## 5. Conclusions

Views of green spaces from home can help to improve the mental health status of the population, in particular, anxiety and depression. The results suggest that adults who do not have access to a green space view from home are associated with a higher risk of anxiety and depression. The study outcomes demonstrate that green views are associated with a lower risk of anxiety and depression.

The elements of nature are not only amenities or a source of pleasure, but essential to well-being and mental restoration. Therefore, a window view, which provides long-term contact with the natural environment, should be considered a key element in urban design.

## Figures and Tables

**Table 1:** Sample of sociodemographic, lifestyle habits, self-perceived health status and Hospital and Anxiety Depression Scale (HADS) in Carmona, Spain.

Sociodemographic Characteristics		Lifestyle Habits, Self-Perceived Health Status and Hads	
Variables	Mean ± SD/*n* (%)	Variables	*n* (%)
Age-group (*N* = 479)	43.0 ± 13.6	Smoker (n = 476), Yes	150 (31.5)
18–31 years	117 (24.4)	Alcohol intake (*N* = 479), Yes	312 (65.1)
32–48 years	168 (35.1)	Physical activity (n = 478), Yes	439 (91.8)
49–64 years	194 (41.0)	View of green spaces from home (n = 478), Yes	116 (24.3)
		Frequency of visits to green spaces (n = 461), Daily	123 (26.7)
Gender (*N* = 479), Males	239 (49.9) 240 (50.1)	No. hours of sleep/day (n = 478)	
Females Marital status (*N* = 479)	239 (49.9) 240 (50.1) 279 (58.2)	<6 h	80 (16.7)
Married		6–7 h	215 (45.0)
Single	157 (32.8)	7–8 h	157 (32.8)
Divorced	21 (4.4)	>8 h	26 (5.4)
Living as a couple	19 (4.0)	Motor impairment (n = 477), Yes	46 (9.6)
Widowed	3 (0.6)	Self-perceived health status (*N* = 479)	
Educational level (n = 478)		Very good	56 (11.7)
Uneducated	60 (13.0)	Good	296 (61.8)
Primary school	214 (45.0)	Regular	91 (19.0)
High school	136 (29.0)	Poor	27 (5.6)
University	69 (14.4)	Very poor	9 (1.9)
Job status (n = 477)		Anxiety—HADS (n = 471)	
Employed	196 (41.1)	No case	281 (59.7)
Unemployed	143 (30.0)	Borderline case	125 (26.5)
Temporary sick leave	17 (3.6)	Case	65 (13.8)
Retired	37 (7.8)	Depression—HADS (n = 466)	
Student	61 (12.8)	No case	423 (90.8)
Homemaker	23 (4.8)	Borderline case	27 (5.8)
		Case	16 (3.4)

HADS, Hospital Anxiety and Depression Scale.

**Table 2:** Association of green spaces views from home with anxiety and depression in Carmona, Spain.

Variables	View of Urban Green Space		
	Yes *n* (%)	No *n* (%)	*p*-Value
**Anxiety (*N* = 470)**			
No case	77 (66.4)	204 (57.6)	0.163
Borderline case	23 (19.8)	101 (28.5)	
Case	16 (13.8)	49 (13.8)	
**Depression (*N* = 465)**			
No case	102 (90.3)	320 (90.9)	0.784
Borderline case	6 (5.3)	21 (6.0)	
Case	5 (4.4)	11 (3.1)	

**Table 3:** Multiple linear regression between anxiety and sociodemographic variables, view of green spaces from home, and frequency of green space visits in Carmona, Spain.

Coefficients					
Model	Non-Standardized Coefficients		Standardized Coefficients	*t*-Score	*p*-Value
	Beta	Deviation Error	Beta		
Age, Older ^1^	0.056	0.013	0.325	4.281	<0.001
Gender, Female ^2^	1.286	0.373	0.115	3.449	0.001
Educational level, High ^3^	0.396	0.187	0.133	2.119	0.035
Marital status, Single ^4^	0.546	0.400	0.095	1.365	0.173
Job status, Unemployed ^5^	0.500	0.358	0.096	1.394	0.164
View of green spaces from home, No ^6^	0.897	0.368	0.207	2.435	<0.015
Frequency of green spaces visits, Daily ^7^	0.057	0.081	0.018	0.708	0.480

^1^ Age variable: from younger to older (18 to 64 years old). ^2^ Gender: reference category female. ^3^ Educational level: from lowest to highest studies (uneducated, primary schooling, high school, and university). ^4^ Marital status: reference category single. ^5^ Job status: reference category unemployed. ^6^ View of green spaces: reference category no view. ^7^ Frequency of green space visits: reference category daily.

**Table 4:** Multiple linear regression between depression and sociodemographic variables, view of green spaces from home, and frequency of green space visits in Carmona, Spain.

Coefficients					
Model	Non-standardized Coefficients		Standardized Coefficients	*t*-Score	*p*-Value
	Beta	Deviation Error	Beta		
Age, Older ^1^	0.012	0.012	0.139	1.011	0.313
Gender, Female ^2^	0.233	0.347	0.041	0.672	0.502
Educational level, High ^3^	−0.200	0.173	−0.133	−1.157	0.248
Marital status, Single ^4^	−0.546	0.370	−0.187	−1.475	0.141
Job status, Unemployed ^5^	1.158	0.334	0.440	3.462	0.001
View of green spaces from home, No ^6^	0.854	0.341	0.389	2.505	0.013
Frequency of green spaces visits, Daily ^7^	0.054	0.076	0.033	0.717	0.474

^1^ Age variable: from minor to major (18 to 64 years old). ^2^ Gender: reference category female. ^3^ Educational level: from lowest to highest studies (uneducated, primary school, high school, and university). ^4^ Marital status: reference category single. ^5^ Job status: reference category unemployed. ^6^ View of green spaces: reference category no view. ^7^ Frequency of green space visits: category reference daily.
